# Identification of Board-Certified Plastic Surgeons Using Artificial Intelligence: An Accuracy Assessment

**DOI:** 10.1093/asjof/ojag123

**Published:** 2026-07-01

**Authors:** Rohun Gupta, Atharva M Bhagwat, Mei Hainline, Herluf Lund, Brian A Mailey, Sumesh Kaswan

## Abstract

**Background:**

Artificial intelligence (AI), particularly large language models like ChatGPT (OpenAI, San Francisco, CA) and Gemini (Google, Mountain View, CA), is increasingly used in healthcare for diagnosis, education, and research. Because interest in cosmetic procedures rises, patients face growing difficulty in selecting qualified providers because of unclear credentials and expanding practitioner types.

**Objectives:**

This study evaluates whether AI can help patients accurately identify board-certified plastic surgeons, addressing a current gap in the literature.

**Methods:**

Four leading AI tools—ChatGPT, Perplexity (Perplexity AI, San Francisco, CA), Gemini, and Claude (Anthropic, San Francisco, CA)—were evaluated by asking each to name board-certified plastic surgeons across all 50 US states. Results were verified through independent internet searches and the American Board of Plastic Surgery to confirm certification status. Statistical analysis using GraphPad Prism (Boston, MA) found differences between tools, with significance set at *α* = .05.

**Results:**

Across 1000 results, overall accuracy in identifying plastic surgeons was 94.1%, with Gemini performing best, followed by Perplexity, ChatGPT, and Claude, with statistically significant differences. Most nonplastic surgeons were otolaryngologists and general surgeons, with no difference between AI models in error type distribution. Gemini scored the highest in regards to board certification accuracy, showing significant differences compared with the other models.

**Conclusions:**

Because the field of aesthetic procedures grows and more providers enter the market, patients must be able to make well-informed choices when selecting a practitioner. Although AI offers a promising tool for identifying board-certified plastic surgeons, its potential for error means patients should independently verify the information.

**Level of Evidence: 5 (Therapeutic):**

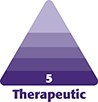

In recent years, there has been a significant growth of artificial intelligence (AI) technology within healthcare.^1^ In particular, AI is able to aid in diagnosis and treatment plan formation, pattern analysis, and education.^2-4^ ChatGPT (OpenAI, San Francisco, CA) was one of the earliest platforms to gain popularity, as evidenced by published literature since 2023.^5,6^ Since the introduction of ChatGPT, there has been a surge in other large language models (LLMs) such as Gemini (Google) and Perplexity.^7,8^ Recent reports have estimated that over 230 million people globally ask health- and wellness-related questions each week to ChatGPT, of which 44% are to learn about their treatment options.^9,10^ Furthermore, countless studies have demonstrated the utility and efficacy of LLMs within fields of medicine, including plastic surgery.

Within plastic surgery, there has been a robust amount of literature that has demonstrated the uses of LLMs. For example, these models have been found to be a versatile tool for resident education by providing general information and promoting case-based learning.^11,12^ Additionally, several LLMs have found to be effective in aiding in the research process, either through grant support or topic conceptualization.^13-16^ Notably, studies have found chatbots to be a valuable resource for managing plastic surgery inquiries and patient education resources.^17-19^

Moreover, several studies, including the 2024 American Society of Plastic Surgeons report, have demonstrated a steady increase in the rate of cosmetic procedures, demonstrating continued consideration by patients.^20,21^ The increased interest in cosmetic procedures has led to an increased number of nonplastic surgeons performing cosmetic procedures.^22^ Furthermore, studies have demonstrated that patients often have difficulty with interpreting the appropriate information to make a selection regarding a provider secondary to marking, unrecognized credentials, and stratification of surgeons.^23^

Given the increased incidence of aesthetic procedures and potential difficulty for patients to adequately select a board-certified plastic surgery provider, it is critical to assess whether AI may play a role in accurately simplifying the process for patients. There is currently a lack of literature that surrounds the use of LLMs for patient aid with regards to surgeon selection. Given this gap in the literature, this study aims to assess whether patients will be available to effectively select board-certified plastic surgeons using LLMs.

## METHODS

Four free-to-public AI models were queried in March 2026: ChatGPT 5.3 (OpenAI), Perplexity Sonar (Perplexity AI, San Francisco, CA), Gemini 3 (Google, Mountain View, CA), and Claude Sonnet 4.6 (Anthropic, San Francisco, CA). Within an incognito search window cleared of cache, cookies, and search history, each was provided with the search request “Provide the names of five board-certified aesthetic plastic and reconstructive surgeons in [*State*]” for each of the 50 states within the United States, and the 5 provided names were collected. This cycle was repeated for each prompt in order to ensure appropriate data collection. Each individual name was then internet searched to verify whether they were a plastic and reconstructive surgeon or another specialty provider. Individuals were then searched through the American Board of Plastic Surgery (ABPS) website to verify whether they were board certified in plastic and reconstructive surgery. A correct output from the AI tool was considered to be a board-certified plastic surgeon as verified by internet search and by ABPS certification status. If not ABPS certified, providers were further characterized by the American Board of Cosmetic Surgery (ABCS), American Board of Medical Specialties (ABMS), or American Board of Facial Plastic and Reconstructive Surgery (ABFPRS).

Statistical analysis was completed using GraphPad Prism 11.0.1 (2026, Boston, MA). Kruskal–Wallis test was used to statistically compare across groups with continuous data, with pairwise comparison and Bonferroni correction for intergroup comparison. Categorical data were assessed using Fisher's exact test. Statistical significance was set at *α* = .05.

## RESULTS

**Figure 1.**
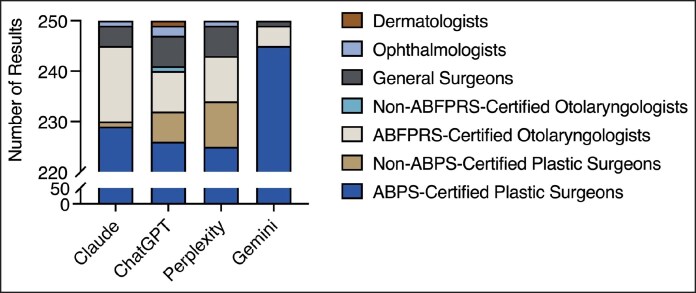
A stacked bar graph detailing distribution of providers identified by artificial intelligence models with significance (*P* = .0136). ABPS, American Board of Plastic Surgery; ABFPRS, American Board of Facial Plastic and Reconstructive Surgery.

A total of 1000 query results were included within this study, 250 for each of 4 AI tools. Zero instances of nonexistent providers were observed across all queries. Accuracy of identifying a plastic and reconstructive surgeon across all 4 models was 94.1%. Gemini had the greatest accuracy at 98.0%, followed by Perplexity at 93.6%, ChatGPT at 92.8%, and Claude at 92.0% (Figure 1). A significant association was found between AI models and identification of a plastic and reconstructive surgeon (*P* = .0098).

Among successfully identified plastic surgeons, 98.3% were found to be board certified by the ABPS. The remaining 1.7% of identified plastic surgeons were trained plastic surgeons that were not ABPS certified. Gemini demonstrated the highest accuracy of identifying ABPS-certified plastic surgeons at 100.0%, followed by Claude at 99.6%, ChatGPT at 97.4%, and Perplexity at 96.2%. A significant relationship was observed between AI models and identification of an ABPS-certified plastic surgeon (*P* = .0009). Overall accuracy of identifying a board-certified plastic and reconstructive surgeon across all 1000 queries was 92.5% across AI models, with Gemini at 98.0%, Claude at 91.6%, ChatGPT at 90.4%, and Perplexity at 90.0%.

Of those that were identified as a specialty other than plastic and reconstructive surgeries, otolaryngologists were most represented at 62.7%, with 97.1% of these surgeons being board certified by the ABFPRS. No significant relationship was observed between AI models and distribution of nonplastic surgeons identified (*P* = .8259). Of all nonplastic surgery providers, 12 out of 60 were associated with the ABCS. Perplexity had the lowest percentage of ABCS-associated providers out of nonplastic surgeons at 14.3% followed by Claude at 17.6%, Gemini at 20.0%, and ChatGPT at 27.8%. No significance was observed for the relationship between AI models and association with ABCS (*P* = .6852). General surgeons comprised 91.7% of ABCS-associated providers, with the remaining 8.3% by ophthalmologists. Notably, 97.1% of otolaryngologists were board certified by the ABFPRS, with each model identifying exclusively ABFPRS-certified otolaryngologists except ChatGPT. However, no significant relationship was observed across AI models (*P* = .5833).

## DISCUSSION

The escalating interest in aesthetic procedures within the United States has led to an expansion in regard to different types of practitioners who offer aesthetic procedures. In several studies, including Chen et al, the authors have demonstrated that despite significant efforts by plastic surgery societies, confusion remains between public perceptions of plastic and cosmetic surgeons.^24^ With the continued integration of AI resources within plastic surgery, this study looked to assess the extent that these resources can help patients find board-certified plastic surgeons.

Although there have been several studies that have examined the role of LLMs within plastic surgery, this is the first study to document the possibility of LLM use by patients for provider selection. Our findings indicate that LLMs may successfully serve as a supplemental tool for patients in finding a board-certified plastic surgeon. Although there were differences between the accuracy of the various models, there was an overall 94.1% accuracy rate determined.

Between the 4 LLMs that were employed, Gemini had the highest accuracy rate, followed by Perplexity, ChatGPT, and Claude. Although there have been no current studies regarding this topic, other AI and plastic surgery related studies have looked to compare the various datasets and have generally found that there may be differences in model accuracy dependent on the study topic. For instance, Köksaldı et al found ChatGPT to be superior in performance compared with Gemini and other LLMs when looking to assess the accuracy of medical content regarding upper blepharoplasty.^25^ In another study, Gomez-Cabello et al found ChatGPT-4 to be more accurate compared with Gemini in providing intraoperative decision making for plastic surgery procedures.^26^

Surgeon reputation and board certification are perhaps some of the most important factors associated with patient selection of plastic surgeons.^27^ Additional factors may include the provider's years of experience with the desired procedure and years in practice.^28^ In the evolving landscape of aesthetic procedures, where various types of physicians provide these services, it is imperative that patients can effectively differentiate among providers. Gelfond et al found that patients typically found plastic surgeons to be the most qualified to perform aesthetic procedures, although there may be growing comfort with other surgical specialties.^29^

Through the 4 LLMs utilized in this study, the overall accuracy of identifying a board-certified plastic and reconstructive surgeon was 92.5%. Of those that were identified as a specialty other than plastic and reconstructive surgeries, otolaryngologists were most represented at 62.7%; 97.1% were board certified ABFPRS. The vast majority of providers analyzed within this study through queries across AI models were board certified by either the ABPS or the ABFPRS. However, a small subgroup of providers claimed board certification from the ABCS and was neither plastic and reconstructive surgeons nor otolaryngologists/head and neck surgeons. Previous literature has demonstrated that ABCS certification does not denote equivalent rigorous specialty surgical training as that by the ABPS or ABFPRS, the latter of which requires prior board certification by either the ABPS or the American Board of Otolaryngology—Head and Neck Surgery.^30,31^ The distinction between plastic surgeons and facial plastic surgeons is contentious within the broader literature as well; however, board certification remains the gold standard for determination of a qualified, rigorously trained aesthetic surgeon.^32,33^ Critically, although AI models largely can aid patients in identifying a board-certified plastic or facial plastic surgeon, patients should verify board certification through the certifying board's own diplomate directory or through state medical board resources, recognizing that ABMS membership and ABMS equivalency are both the most meaningful indicators of rigorous credentialing.

There are several notable limitations that must be addressed. In order to improve generalizability of our study, we attempted to include 5 providers from each of the 50 states. In doing so, there are a limited number of practitioners that are assessed, which may skew results. Additionally, there are inherent differences between the LLMs, including Gemini being directly linked into Google search, allowing for real-time information and fact-checking, which may confound results. Additional differences may include that Perplexity tends to pull data that are verified, while Gemini also interprets material. Also, this study utilized a singular specific prompt, which was detailed in order to increase the probability of obtaining a plastic surgery provider. Given this, it is likely that additional prompts with less specifications would result in vastly different results. Furthermore, the specific prompt that was queried for this study may not completely reflect what an actual patient may search, which may further limit the generalizability of these results. Lastly, AI models are constantly being updated, which may cause the data obtained from each of these models to change.

## CONCLUSIONS

In the expanding landscape of aesthetic procedures and providers, it is critical that patients make informed decisions regarding practitioner selection. AI has emerged as a potentially valuable tool for identifying board-certified plastic surgeons; however, its susceptibility to error necessitates additional patient verification. In order to combat this, patients should consider performing adequate research regarding their provider through the utility of the ABPS website.
